# Impact and legacy of the highly cited paper by Blaxter and Clapperton (1965) ‘Prediction of the amount of methane produced by ruminants [*Br J Nutr* 19, 511–522]’

**DOI:** 10.1017/S0007114522000678

**Published:** 2022-06-28

**Authors:** R. John Wallace

**Affiliations:** Rowett Institute, University of Aberdeen, Aberdeen AB25 2ZD, UK

**Keywords:** Cattle, Methane, Sheep

## Abstract

The paper by K. L. Blaxter and J. L. Clapperton (1965) ‘Prediction of the amount of methane produced by ruminants. *Br J Nutr* 19, 511–522’ has been cited 656 times according to Web of Science and continues to be cited with increasing frequency to the present day. The analysis described in the paper, or meta-analysis as it would be known now, is of methane production from cattle and sheep based on forty-eight trials using closed-circuit respiration chambers, all carried out at the Hannah Research Institute, Ayr, UK, between 1955 and 1965. Methane emissions per unit of diet fed were shown to vary depending on diet, level of feeding and individual animal. As such, previous notions that methane emissions were essentially proportional to energy intake were set aside. The main reasons for the paper’s continuing citation are the set of equations that can be used to predict methane emissions from ruminants when the technically demanding respiration chambers are unavailable, and that it was the first definitive study to describe the complexities of methane emissions with respect to animals and diets. The paper thus provided abundant insights of the relations between ruminant methane emissions and nutritional biology, and rumen microbiology, in particular, that have informed countless research projects in the intervening half-century. Given the importance of methane as a greenhouse gas in the climate change scenario, these insights are at least as relevant today as they were in 1965.

Methane emission from ruminant livestock is a topic that has interested scientists for many decades. At the time of the Blaxter and Clapperton (1965) paper, the emphasis was on completing inventories of energy intake and expenditures of farm livestock by making the challenging measurements of methane production by individual animals and relating those to feed intake. Microbiologists were also fascinated by the methanogenic ‘bacteria’^(1)^ (now known as archaea, in size and shape rather similar to bacteria but with different metabolic properties and evolutionary origins^(2)^) responsible for methane production. In contrast to those rather niche interests, present-day interest stems from the realisation that the well-being of humanity depends in part on lowering greenhouse gas emissions, of which ruminant livestock contribute very significantly^(3)^. Consequently, the emphasis of current research efforts is to lower methane emissions, whether by chemical, genetic or other means^(4–6)^.

Ruminant animals, including cattle, sheep and goats, are among the most efficient herbivores on earth, by virtue of their gut anatomy and high microbial activity in the foregut compartment – the reticulorumen, or rumen for short^(7)^. Fibrous plant materials are broken down by microbial activity to SCFA, principally acetate, propionate and butyrate, that are absorbed from the gut and used by the host animal for energy and growth^(8,9)^. Methane is a consequence of the anaerobic nature of the rumen fermentation, as it is in other anaerobic habitats^(10)^. A complete account of the nutritional energetics of ruminants could not be achieved without the precise quantitation of methane emissions.

## What’s in the paper?

**Fig. 1.**
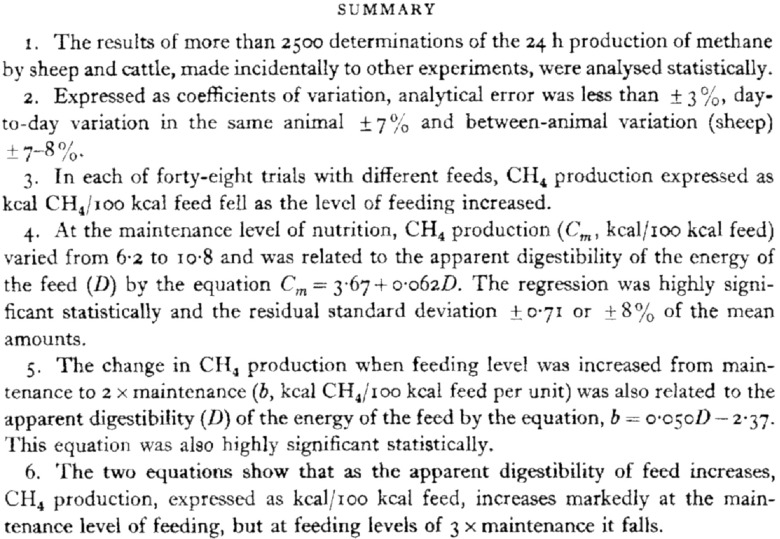
The original summary.

The Summary (Fig. 1) describes the results of the meta-analysis, in terms of analytical error, animal and time variations and the effects of nutrient intake on methane emissions. It also refers to the dependence of methane production on the apparent digestibility of the feed and the intake level relative to maintenance requirements, which impact heavily on contemporary questions about how ruminants should be fed, in terms of dietary constituents and feeding practices, to minimise the environmental impact of livestock production. The paper begins with the context of the work at the time:

‘Metabolisable energy is defined as the heat of combustion of a feed less the heat of combustion of the faeces, urine and gases which are produced when it is eaten. The losses of energy in faeces and urine can be determined easily in sheep and cattle kept in metabolism cages, but to determine the energy they lose as combustible gas, that is as methane, involves quantitative measurement of the gaseous exchange and the use of much more complex and expensive equipment’. Regarding the latter, it is instructive to note that the authors regarded a thermal conductivity method following combustion of methane in samples as state of the art, in contrast to the simple method of choice today, GC^(11)^.

The conclusions of the paper are wrapped in metabolic equations, but the main impact on the reader derives from the figures, in which the effects of diet type, feeding level and apparent digestibility are clearly illustrated. The results showed clear and sustained inter-animal and dietary differences that had been suspected before but were now demonstrated with commanding clarity.

## What is its citation history?

**Fig. 2.**
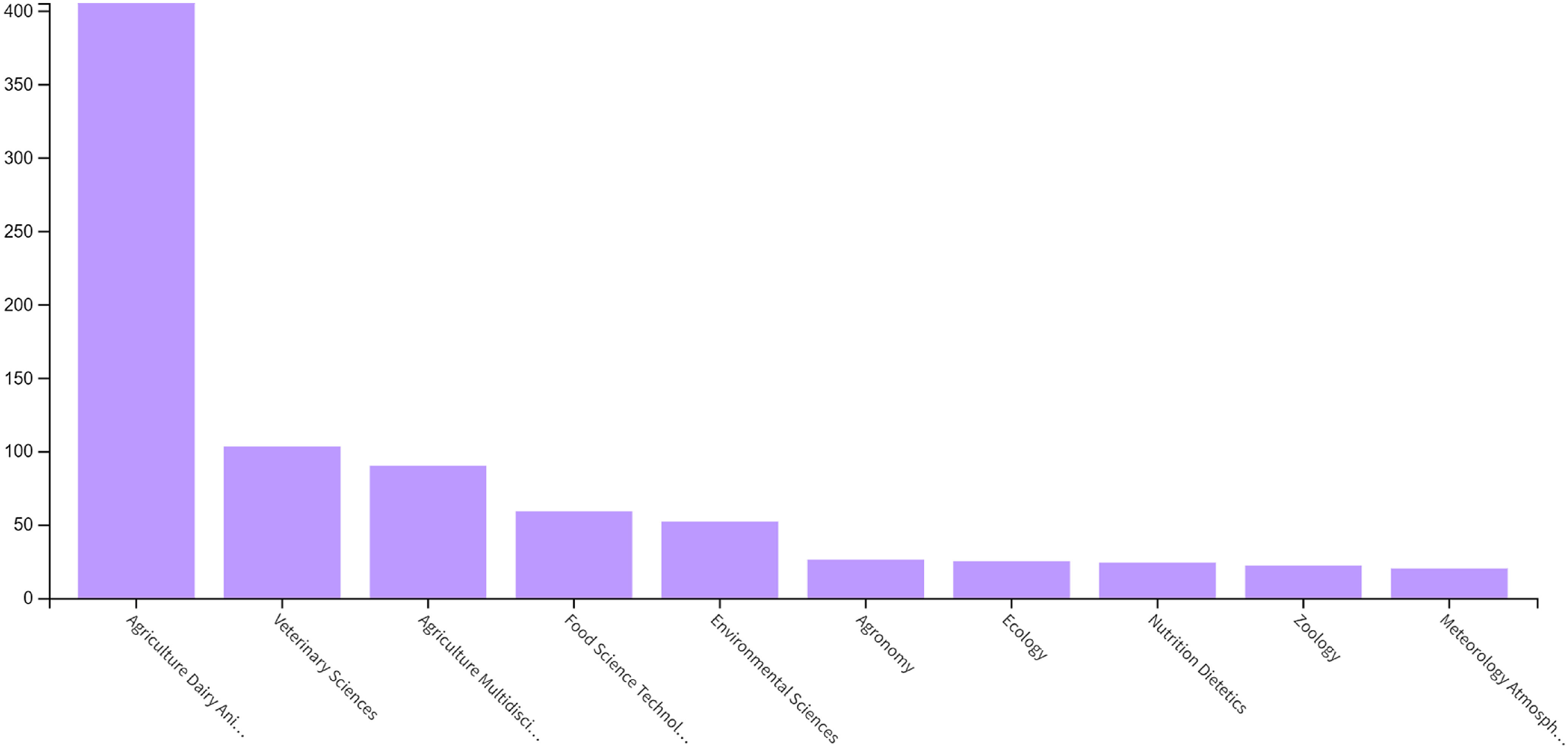
Subject areas of citations as determined by web of science.

**Fig. 3.**
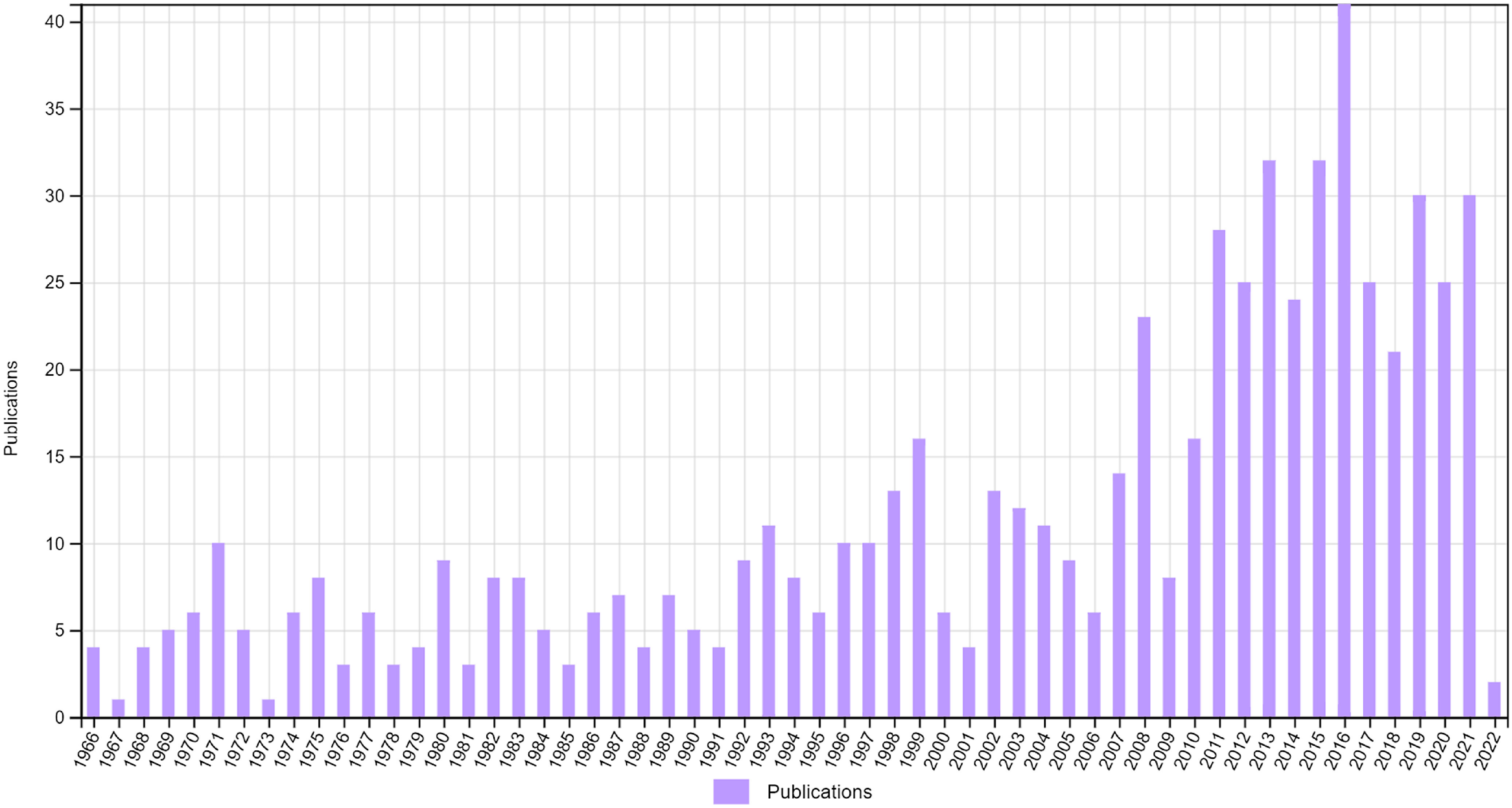
Annual citation rate as determined by web of science.

According to Web of Science, the paper has received 656 citations, of which 405 were categorised as Agriculture, Dairy and Animal Science, 52 were Environmental Sciences, 25 Ecology, 24 Nutrition and Dietetics and 59 Food Science Technology (Fig. 2). Thus, the paper has had wide impact across biological and social sciences. The trend for the number of publications citing Blaxter and Clapperton (1965) is upwards as years pass (Fig. 3), rather than to decline as one might expect for most papers. The trend shows no real sign of declining.

## Why has the paper been cited so frequently?

Papers published in 2018 were scanned for the Blaxter and Clapperton (1965) citation to investigate whether the authors were citing a single technical property of the paper (e.g. as in the classical Lowry method of protein assay^(12)^) or if other more fundamental observations were being replicated. Twenty-one papers were identified by Web of Science, of which seventeen were open access (Table 1). The most common reason for the citation was that Blaxter and Clapperton (1965) was the first demonstration that methane emissions varied with the amount of dietary intake, but not in a simple manner. Others cited one of the equations in the original paper, or noted that respiration chambers were used, or were general citations about methane emissions from livestock. The spread of journals mostly reflected the direct relevance to animal research. However, several more environmentally orientated journals appeared also. Thus, several different features of the paper were cited by researchers from a range of disciplines, not only animal scientists.

**Table 1.** Open access papers citing Blaxter and Clapperton (1965) published in 2018

Authors	Journal	Reason for citation
Amer *et al.*	*Animal* **12**, 5–11	Methane emission linked to feed intake
Bharanidharan *et al.*	*PLOS One* **13**, e0202446	Relation between emissions and feed intake
Darcan & Silanikove	*Small Rum Res* **163**, 34–38	Equation connecting intake, digestibility and level of feeding
Eckert *et al.*	*Land* **7**, 26	Use of respiration chambers
Fernandez *et al.*	*Glob Ecol Conserv* **15**, e00439	General citation of meta-analysis
Hansen *et al.*	*Polar Res* **37**, 1505396	Citing equations connecting intake, digestibility and level of feeding; animal and diet effects
Hristov *et al.*	*J. Dairy Sci* **101**, 6655–6674	Review; nature of equations, dependence on feed type
Kennedy *et al.* a,b	*J Agric Sci* **156**, 1005–1016a, 1017–1027b	Use of factor relating methane emission to energy intake
Kidane *et al.*	*J Anim Sci* **96**, 3967–3982	General, emissions related to intake
Ku-Vera *et al.*	*Agric For Meteorol* **258**, 3–7	Equations relating emissions and feed intake
Liu *et al.*	*PLOS One* **13**, e203393	Extrapolation of energy calculations to rabbits
Mombach *et al.*	*Braz J Anim Sci* **47**, e20170190	Use of respiration chambers
Na *et al.*	*Asian-Australas J Anim Sci* **31**, 1238–1243	Relation between emissions and feed intake
Wu *et al.*	*J Dairy Sci* **101**, 1554–1564	Between-animal variability in respiration chambers
Xiang *et al.*	*Front Genet* **9**, 330	Relation between emissions and feed intake
Zagorakis *et al.*	*S Afr J Anim Sci* **48**, 489–496	Use of equations

## Personal note

Kenneth Blaxter moved from the Hannah Research Institute in 1965 to become Director of the Rowett Research Institute in Bucksburn, just outside Aberdeen. There he constructed similar respiration chambers to those at the Hannah, and thus, he continued his work on the energy metabolism of ruminants. Under his Directorship, the Rowett gained international renown for research on the nutrition of farm livestock, including red deer. He was made FRS in 1967 and was knighted for his contribution to science in 1977. Kenneth Blaxter was Director of the Rowett when I was appointed in 1976. Blaxter was an inspirational man; he had a fierce intellect, loving to draw together information and data to tell a coherent story. I well remember entering his office sometime in the late 70s to find him doodling about the impact of forestry on the global carbon economy – amazingly prescient given today’s climate crisis. The featured paper with John Clapperton exemplifies his legacy to nutritional science.
